# Curcumin Supplementation (Meriva^®^) Modulates Inflammation, Lipid Peroxidation and Gut Microbiota Composition in Chronic Kidney Disease

**DOI:** 10.3390/nu14010231

**Published:** 2022-01-05

**Authors:** Francesca Pivari, Alessandra Mingione, Giada Piazzini, Camilla Ceccarani, Emerenziana Ottaviano, Caterina Brasacchio, Michele Dei Cas, Margherita Vischi, Mario Gennaro Cozzolino, Paolo Fogagnolo, Antonella Riva, Giovanna Petrangolini, Luigi Barrea, Laura Di Renzo, Elisa Borghi, Paola Signorelli, Rita Paroni, Laura Soldati

**Affiliations:** 1 Biochemistry and Molecular Biology Laboratory, Department of Health Sciences, Università degli Studi di Milano, 20142 Milan, Italy; alessandra.mingione@unimi.it (A.M.); giada.piazzini@studenti.unimi.it (G.P.); paola.signorelli@unimi.it (P.S.); 2 Institute of Biomedical Technologies, Italian National Research Council, 20090 Segrate, Italy; camilla.ceccarani@itb.cnr.it; 3 Laboratory of Microbiology, Department of Health Sciences, Università degli Studi di Milano, 20142 Milan, Italy; emerenziana.ottaviano@unimi.it (E.O.); elisa.borghi@unimi.it (E.B.); 4 Department of Health Sciences, Università degli Studi di Milano, 20142 Milan, Italy; caterina.brasacchio@unimi.it (C.B.); mario.cozzolino@unimi.it (M.G.C.); paolo.fogagnolo@unimi.it (P.F.); 5 Clinical Biochemistry and Mass Spectrometry Laboratory, Health Sciences Department, Università degli Studi di Milano, 20142 Milan, Italy; michele.deicas@unimi.it (M.D.C.); rita.paroni@unimi.it (R.P.); 6 Nephrology and Dialysis Unit, A.S.S.T. Santi Paolo e Carlo, 20142 Milan, Italy; margherita.vischi@asst-santipaolocarlo.it; 7 Ophthalmology Unit, A.S.S.T. Santi Paolo e Carlo, 20142 Milan, Italy; 8 R & D Indena S.p.A, 20139 Milan, Italy; antonella.riva@indena.com (A.R.); giovanna.petrangolini@indena.com (G.P.); 9 Dipartimento di Medicina Clinica e Chirurgia, Unit of Endocrinology, Federico II University Medical School of Naples, 80131 Naples, Italy; luigi.barrea@unina.it; 10 Section of Clinical Nutrition and Nutrigenomic, Department of Biomedicine and Prevention, University of Rome Tor Vergata, 00133 Rome, Italy; laura.di.renzo@uniroma2.it

**Keywords:** chronic kidney disease, curcumin, inflammation, lipid peroxidation, gut microbiota, uremic toxins, Meriva

## Abstract

Chronic kidney disease (CKD) subjects suffer from high risk of cardiovascular mortality, and any intervention preventing the progression of CKD may have an enormous impact on public health. In the last decade, there has been growing awareness that the gut microbiota (GM) can play a pivotal role in controlling the pathogenesis of systemic inflammatory state and CKD progression. To ameliorate the quality of life in CKD subjects, the use of dietary supplements has increased over time. Among those, curcumin has demonstrated significant in vitro anti-inflammatory properties. In this pilot study, 24 CKD patients and 20 healthy volunteers were recruited. CKD patients followed nutritional counselling and were supplemented with curcumin (Meriva^®^) for six months. Different parameters were evaluated at baseline and after 3–6 months: uremic toxins, metagenomic of GM, and nutritional, inflammatory, and oxidative status. Curcumin significantly reduced plasma pro-inflammatory mediators (CCL-2, IFN-γ, and IL-4) and lipid peroxidation. Regarding GM, after 6 months of curcumin supplementation, *Escherichia-Shigella* was significantly lower, while *Lachnoclostridium* was significant higher. Notably, at family level, *Lactobacillaceae* spp. were found significantly higher in the last 3 months of supplementation. No adverse events were observed in the supplemented group, confirming the good safety profile of curcumin phytosome after long-term administration.

## 1. Introduction

Chronic kidney disease (CKD) is a pathological condition due to the progressive loss of renal function. From early stages to the end-stage renal disease (ESRD), CKD reaches 13.4% of worldwide prevalence [1]. Global increasing incidence and prevalence of CKD are reasons for concern. CKD is widely recognized as one of the most relevant risk factors for developing cardiovascular diseases (CVD) [2], which is, in turn, the leading cause of morbidity and mortality for dialysis patients [3]: the increasing CKD incidence, then, is related not only to a projected greater need for renal replacement therapy (RRT), but also to an expected increased incidence of CVD. In this context, any medical or dietetic intervention preventing the progression of CKD towards ESRD and improving the patients’ cardiovascular status may have an enormous impact on a public health level. Among the pathogenic factors for the progression of CKD and the occurrence of its main complications, inflammation and oxidative stress have been claimed to play a major role [4]. Moreover, it is well known that in ESRD, a spontaneous reduction of protein and caloric intake often occurs: the “Modification of Diet in Renal Disease Study Group” [5] study showed that patients with ESRD had a protein and caloric intake reduction with progressive decline in renal function, a phenomenon even more pronounced in the elderly. It is also known that malnutrition, linked to inflammation and oxidative stress, is a risk factor associated with increased morbidity and mortality of patients on hemodialysis [6]. Several interventions to delay the progressive loss of renal function and/or to prevent the development of CVD have been suggested and are now used by nephrologists. These include blood pressure and proteinuria control; correction of calcium-phosphate disorders and anemia; smoking cessation, and low-protein diets [7]. 

The growing awareness that the gut microbiota (GM) could play a pivotal role in controlling a number of homeostatic host functions [8] gave purpose to researchers to explore its possible connections to CKD. Indeed, the changes in quantitative and/or qualitative composition of the intestinal microbial population have been implicated in the pathogenesis of different illnesses, including systemic inflammatory state, CKD progression, and CKD-related cardiovascular complications [9]. It has been observed that GM consistently changes over the course of CKD, inducing a metabolic burden that could further increase the cardiovascular risk of CKD patients [10]. Moreover, metabolites derived from GM, including the fermentation products of proteins or choline, such as p-cresyl sulfate (PCS) and indoxyl sulfate (IS), may contribute to decline kidney function and worsen CVD [11].

While the human body naturally undergoes a series of changes in the immune system and the oxidative status over its lifespan, CKD patients are also characterized by a persistent state of low-grade chronic inflammation. This inflammation-mediated aging, called “inflammaging”, is emerging as a central patho-mechanism of aging, suggesting that it may be modulated to reduce the burden of disease in the elderly [12]. One of the possibilities to modulate the systemic inflammatory condition is to act on the composition of the GM. It has been suggested that interventions directed to modulate the bacterial species inhabiting the gastrointestinal tract through supplementation with either probiotics/prebiotics or nutraceuticals might be effective in modifying the clinical outcomes directly or indirectly mediated by changes in the inflammatory and oxidative status [13,14]. Referring to patients affected by renal disease, however, the majority of scientific evidence has been obtained in experimental animal models or in the advanced stages of CKD, with few data produced in the earlier stages of the disease, when the intervention might be more plausibly effective [15].

To ameliorate the quality of life in unhealthy subjects, the use of dietary supplements has recently and steadily increased. Among those, curcumin, an active ingredient in the traditional herbal remedy and dietary spice turmeric (*Curcuma longa*), has demonstrated significant in vitro anti-inflammatory properties [16]. Recent studies have shown that curcumin increases the expression of intestinal alkaline phosphatase and tight junction proteins, correcting gut permeability and thus reducing the levels of circulatory inflammatory biomolecules. Therefore, curcumin has potential anti-inflammatory in vivo effects on CKD [17,18]. 

A wide range of curcumin formulations have been explored, through the years, in order to improve the poor bioavailability and low oral absorption of curcumin. The food-grade formulation of curcumin in phospholipids (Meriva^®^) improved curcumin bioabsorption thanks to the lecithin phospholipids delivery system (Phytosome^®^) able to optimize the bioabsorption of the extract, with a physical and not pharmacological mechanism, preventing curcumin self-aggregation [19]. Moreover, curcumin phytosome showed a more efficient curcuminoids biotransformation by the human gut microbiota compared to unformulated curcumin, without altering the natural profile of curcuma metabolites [20]. This suggests a potential improved clinical efficacy, since curcuminoid-reduced metabolites have been recently reported as biologically active molecules in scientific literature (e.g., anti-inflammatory, antioxidant, hepato-protective effects). In a preclinical study, where the mechanism of action of this formulation was deeply explored, curcumin phospholipids proved to have a triple effect, being hepatoprotective, anti-inflammatory, and chemopreventive [21]. Noteworthy is that curcumin phytosome allowed the exploitation of curcumin’s beneficial activities in several unhealthy conditions, keeping a good safety profile, as reported in human studies [22,23,24,25,26]. The anti-inflammatory properties were also noted by a preliminary human study in which curcumin phytosome exhibited positive effects on kidney health in subjects with temporary kidney disfunction, by ameliorating both objective biomarkers (e.g., reduced albuminuria and oxidative stress) and subjective symptoms (e.g., individuals’ fatigue) [27].

In this scenario, our study aimed to observe the potential benefits of the oral curcumin–phospholipid supplementation in subjects with mild CKD (stages 3a–4) through several investigations. Along with the assessment of the supplementation safety, we explored its interaction between the nutritional, inflammatory, and oxidative statuses from a clinical point of view, and then focused on the gut microbiota composition and its changes due to CKD and curcumin intake. 

## 2. Materials and Methods

### 2.1. Patients

We enrolled 24 chronic kidney disease patients (CKD group) in conservative therapy at the Nephrology and Dialysis Unit of the Azienda Socio-Sanitaria (A.S.S.T.) Santi Paolo and Carlo, recruited from February 2018 to February 2019. In order to participate in the study, patients had to meet the following inclusion criteria: CKD from stage 3a to 4 (defined according to the GFR values of the KDOQI guidelines [7]), not being on hemodialysis treatment; age ≥ 18 years; absence of chronic infections, active neoplasm, vasculitis, autoimmune or acute inflammatory diseases, gastro-intestinal pathologies, dementia, steroid therapies, and pregnancy. Drop-out rates, due to reasons not related to curcumin supplementation, were 12.5% (*n* = 3) after 3 months and 47.6% (*n* = 10) after 6 months. No adverse effects were reported.

The age- and sex-matched control group (20 subjects) was enrolled at the Ophthalmology Unit of the A.S.S.T. Santi Paolo and Carlo (Milan, Italy), in particular for anthropometric, nutritional, and microbiome analyses. In order to participate in the study, control subjects had to meet the same above-mentioned inclusion criteria, except for the presence of CKD or other kidney-related pathologies.

All subjects gave their informed consent for inclusion before enrollment. The study was conducted in accordance with the Declaration of Helsinki, and the protocol was approved by the Ethics Committee of Milano Area 1 (MRC Project: 2017/ST/035). The study was registered to ISRCTN registry (ISRCTN10446067).

### 2.2. Study Design and Supplementation

**Scheme 1:**
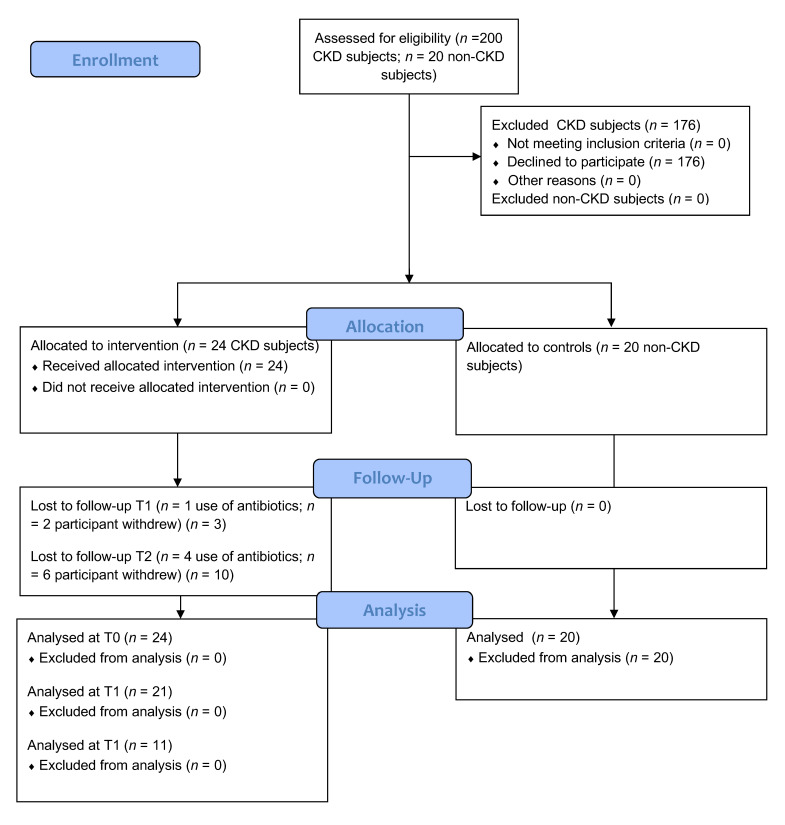
CONSORT flow diagram. CKD patients, chronic kidney disease patients; T0, baseline; T1, after 3 months; T2, after 6 months.

CKD patients (*n* = 24) received Meriva^®^ 500 mg/tablet twice in a day for 3 or 6 months. Meriva^®^ was supplied by INDENA S.p.A. (Milan, Italy) as a food-grade lecithin formulation of curcumin in 500 mg film-coated tablets, containing a standardized amount of 100 mg highly bioavailable curcuminoids. At baseline (T0) and after 3 months (T1) and 6 months (T2) of Meriva^®^ supplementation we collected patients’ clinical parameters, anthropometric and body composition measures, dietary habits, and stool and blood samples. In Scheme 1, the CONSORT flow diagram of the study is shown.

### 2.3. Clinical Parameters

Clinical data routinely investigated in the patient through periodic nephrological visits were collected. In particular, we considered the following clinical parameters: serum creatinine (mg/dL), azotemia (mg/dL), sodium (mEq/L), potassium (mEq/L), calcium (mg/dL), phosphorous (mg/dL), and GFR (CKD–EPI formula).

### 2.4. Body Composition

At each timepoint, the following anthropometric measures were collected: weight (kg), height (m), waist circumference (cm), body mass index (BMI, calculated as body weight expressed as kilograms divided by squared height reported in meters). Body composition was assessed by bioimpedance analysis (BIA) through Body Composition Monitor (Fresenius Medical Care Italia S.p.A., Palazzo Pignano, Italy), mainly including total body water (TBW), extracellular water (ECW), intracellular water (ICW), fat-free mass (FFM), and fat mass (FM).

### 2.5. Dietary Habits and Nutritional Counseling

A three-day food diary was used to estimate patients’ food consumption. During the nutritional visit, nutritionists, through the use of the Scotti Bassani Photographic Atlas to better estimate food portions, analyzed the diary of the patient. The 3-day food diary is a method of nutritional investigation, validated in the literature [28], that provides for the recording of all foods and drinks consumed daily, during a period of time of 3 days, including 2 working days and one during the weekend.

We used Ephood^®^ (Sanipedia S.r.l., Bresso, Italy) web platform to collect patients’ nutritional data, dietary micronutrients and macronutrients. Ephood^®^ software is based on the food database of the European Institute of Oncology and contains 1570 servings, 830 foods, and 40 nutrients. During the first visit (T0), patients received nutritional counseling to ameliorate dietary habits and improve conservative therapy, in accordance with the KDOQI guidelines [7].

### 2.6. Inflammation and Lipid Peroxidation

The inflammation level was evaluated on patients’ plasma samples (blood samples centrifuged at 4000 rpm at 4 °C for 20 min). In particular, eight cytokines/chemokines (IL-1β, IL-4, IL-6, IL-12p70, IL-18, MCP-1, TNF-α, and INF-γ) were measured using the ELISA (enzyme-linked immunosorbent assays) immunological test. We used the Fluorokine^®^ Multianalyte Profiling kit (R & D Systems, Minneapolis, MN, USA) through the Luminex^®^ 200 analyzer, according to the manufacturing procedures. 

To assess lipid peroxidation level in plasma samples, a Parameter™ TBARS (thiobarbituric-acid-reactive substances) assay was used (R & D Systems, Minneapolis, MN, USA), according to the manufacturing procedures. Briefly, in the presence of heat and acid, malondialdehyde (MDA) reacts with TBA to produce a colored end-product that absorbs light at 530–540 nm. The intensity of the color at 532 nm corresponds to the level of lipid peroxidation in the sample, measured through a multimode microplate reader, EnSight™ (PerkinElmer, Beaconsfield, UK). 

### 2.7. LC–MS/MS Analysis of Uremic Toxins 

For the determination of total amount of uremic toxins, plasma (100 µL) was added with IS (25 µL of Indoxyl ^13^C_6_ sulfate 50 µM) and deproteinized with 300 µL of 0.1% formic acid in acetonitrile. After centrifugation (5 min, 13,400 rpm), 60 µL of the clean extract was diluted with 100 µL of ammonium formate buffer (5 mM) and 10 µL was injected in the LC–MS/MS apparatus. 

For the determination of free circulating uremic toxins, plasma (200 µL) was added with IS (50 µL of Indoxyl ^13^C_6_ sulfate 5µM in formate ammonium buffer 25 mM) and centrifuged in a Vivaspin 500 30K MWCO PES for 25 min at 12,000 rpm. The protein-free extract (200 µL) was withdrawn in a vial, and 10 µL injected in the LC–MS/MS apparatus. 

LC–MS/MS conditions: the analytical system consists of an HPLC Dionex 3000 UltiMate system (Thermo Fisher Scientific, Waltham, MA, USA) coupled to a tandem mass spectrometer AB Sciex 3200 QTRAP (Sciex, Milan, Italy) operated under negative ESI mode. The analytes were examined by multiple reaction monitoring, checking the transitions as follows: indoxyl ^13^C_6_ sulfate *m/z* 218 > 80 (DP −40 eV; CE −34 eV), indoxyl sulfate *m/z* 212 > 80 (DP −40 eV; CE −30 eV), and p-cresyl sulfate *m/z* 187 > 80 (DP −40 eV; CE −38 eV). The instrument parameters were as follows: CUR 30, GS1 40, GS2 50, capillary voltage −4.5 kV, and source temperature 550 °C. Chromatographic separation was achieved on an Acquity UPLC CSH Fluoro-phenyl 1.7 µm 2.1mm × 100 mm column (Waters, Milford, MA, USA) using as mobile phase of (A) ammonium formate 5 mM + 0.01% formic acid, and (B) acetonitrile. The elution program (%B) was 0–4 min 20–50%, 4–4.5 min 50–20%, maintained until 10 min. The flow rate was 0.35 mL/min, and the column and the autosampler temperatures were 30 °C and 10 °C.

### 2.8. Gut Microbiota Sequencing

DNA was extracted from 200 mg of each stool specimen using the PSP^®^ Spin Stool DNA Plus Kit (Stratec Biomedical, Birkenfeld, Germany) according to the manufacturer’s instructions. Extracted DNA was quantified by NanoPhotometer^®^ NP80 (Implen, Munich, Germany), and the acceptable concentration was >50 ng/L. The samples were stored at −20 °C until analysis. 

Intestinal bacterial genome sequencing was performed through the next-generation sequencing (NGS), using the Ion 16S™ Metagenomics Kit (Thermo Fisher Scientific, Waltham, MA, USA) designed for rapid analysis of polybacterial samples and capable of analyzing mixed microbial populations by sequencing 7 (V2, V3, V4, V6, V7, V8, and V9) of the 9 hypervariable regions of the rRNA 16S. The amplified fragments were sequenced through Ion PGM™ Systems (Thermo Fisher Scientific, Waltham, MA, USA), according to the manufacturer’s instructions, and then analyzed through Ion Reporter™ software. 

### 2.9. Statistical Analysis

The quantitative variables are reported as average values ± standard deviation. Statistical analyses of the clinical parameters were performed using GraphPad Prism 6 software for MacOs version. 

Body composition analysis and blood chemistry tests were described using the Shapiro–Wilk W test for normal data to compare the characteristics of the CKD population at baseline (T0), after 3 months (T1), and after 6 months (T2) from the start of curcumin supplementation. Comparisons among groups were performed through paired T-test for parametric data and Wilcoxon signed-rank test for nonparametric data. Plasma uremic toxins, cytokines, and lipid peroxidation were analyzed using one-way ANOVA with Bonferroni correction. As for the gut microbiota data, the obtained 16S rRNA gene-paired sequences were merged using Pandaseq (release 2.5) [29]. Reads were filtered by trimming stretches of 3 or more low-quality bases (quality < 3) and discarding the trimmed sequences whenever they were shorter than 75% of the original one. 

Bioinformatic analyses on gut microbiota were conducted using the QIIME pipeline (release 1.9.0) [30], clustering filtered reads into operational taxonomic units (OTUs) at 97% identity level and discarding singletons as possible chimeras. Taxonomic assignment was performed via the RDP classifier [31] against the SILVA database (release 132) [32]. 

Alpha-diversity was computed through the QIIME pipeline using the Chao1, the number of OTUs, Shannon diversity, and Faith’s phylogenetic diversity whole tree (PD whole tree) metrics; statistical evaluation among alpha-diversity indices was performed by a nonparametric Monte Carlo-based test. To compare the microbial community structure of the subjects, weighted and unweighted UniFrac distances and the Permanova test (adonis function) in the R package vegan (version 2.0–10) [33] were used. Statistical taxonomic differences were established through the R package “rstatix” (R version 3.6.3 via RStudio, version 1.2.1335) with the Bonferroni correction. *p*-values below 0.05 were considered significant among all comparisons and analyses, and labelled in the text either raw and/or adjusted for clarity.

## 3. Results

### 3.1. Patients’ Characteristics: Anthropometric Data and Clinical Parameters

In the present study, 24 patients affected by chronic kidney diseases were recruited, (CKD group). Of these, 58% (*n* = 14) were male and 42% (*n* = 12) were female, mean age 71 ± 12 years old. Patients were stratified by CKD staging as follows: 20.8% (*n* = 5) were stage 3a; 33.4% (*n* = 8) were stage 3b, and 45.8% (*n* = 11) were classified as stage 4.

As a control (CTRL) group, we enrolled 20 subjects without CKD, age- and gender-matched. Within the CTRL group, 60% (*n* = 12) were male and 40% (*n* = 8) were female, mean age 73 ± 8 years old. The absence of chronic kidney disease was confirmed by creatininemia and GFR values, statistically different from the CKD groups (Table 1).

Table 1 summarizes cohort characteristics, anthropometric data, bioimpedance analysis, and clinical parameters. For the CKD group, parameters at the baseline (CKD T0), after 3 months (CKD T1), and 6 months (CKD T2) of Meriva^®^ supplementation are reported.

**Figure 1:**
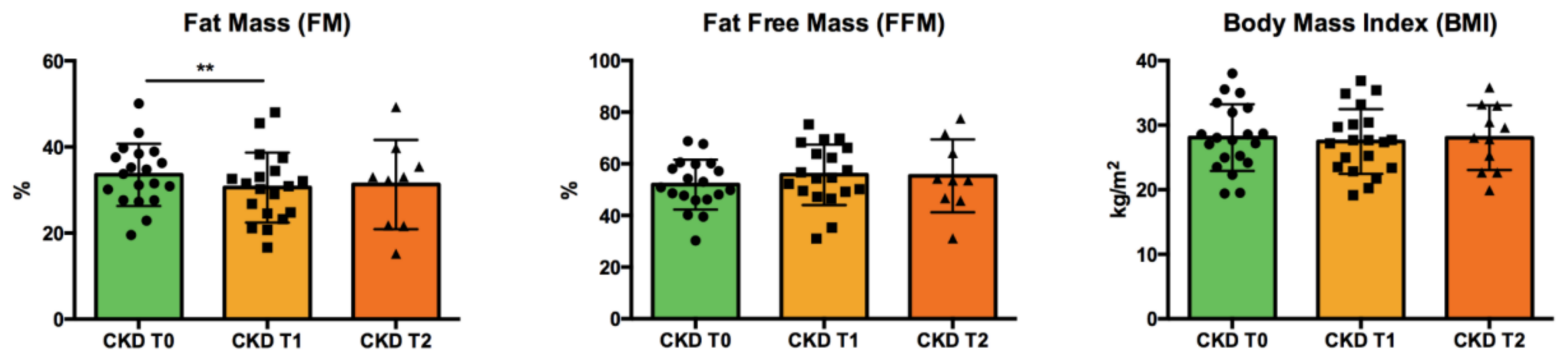
Effects of Meriva^®^ supplementation on fat mass (FM), fat-free mass (FFM), and BMI: comparison between baseline (CKD T0), after 3 months supplementation (CKD T1), and after 6 months supplementation (CKD T2); ** *p* < 0.001.

No significant differences were found for gender distribution, age, BMI, and waist circumference between controls and CKD patients at the enrollment (T0) (Table 1). The bioimpedance analysis showed for the CKD group a significant reduction in fat mass (FM) percentage (T0 vs. T1; *p* < 0.0001) and a positive, but not significant, increasing trend in fat-free mass (FFM) percentage after three months of Meriva^®^ supplementation, at time T1 (Figure 1). No other differences in anthropometric parameters were observed at time T2, after six months of Meriva^®^ supplementation. 

### 3.2. Nutrients Intake

At enrollment, nutritional counseling was provided to participants. However, the majority of patients were already familiar with the nutritional indications, as diet is an integral part of their conservative therapy [7]. 

The quantity and quality of macronutrients and micronutrients were extrapolated from the three-day food diary (Table 2). Nutrients’ intake was analyzed at time T0, T1, and T2 and also compared to the dietary habits reported by the CTRL group (Table 2). Results showed a significant reduction in daily energy intake during the first trimester (CKD T0 vs. T1, *p* < 0.05). At baseline, it is shown that CKD patients consumed more carbohydrates than the CTRL population did (*p* < 0.05). This amount of total carbohydrates significantly decreased after three months (*p* < 0.01) and after six months (*p* < 0.001) of curcumin supplementation. In particular, a reduction was observed in starch intake at time T1 (*p* < 0.05) as well as at time T2 (*p* < 0.01), and a decrease in soluble carbohydrates during the entire semester (*p* < 0.05). A significant decrease in total protein consumption at time T1 (*p* < 0.05) was also observed. A more detailed analysis showed a reduction in plant proteins consumption after 3 and 6 months (*p* < 0.01). Instead, no significant differences were reported in lipids intake. Moreover, the amount of total fiber intake significantly decreased at time T1 (*p* < 0.05). The principal micronutrients intake data showed a reduction in the consumption of phosphorus and potassium after three months (*p* < 0.01), significantly lower than the CTRL group intakes (*p* < 0.05).

### 3.3. Inflammation

**Figure 2:**
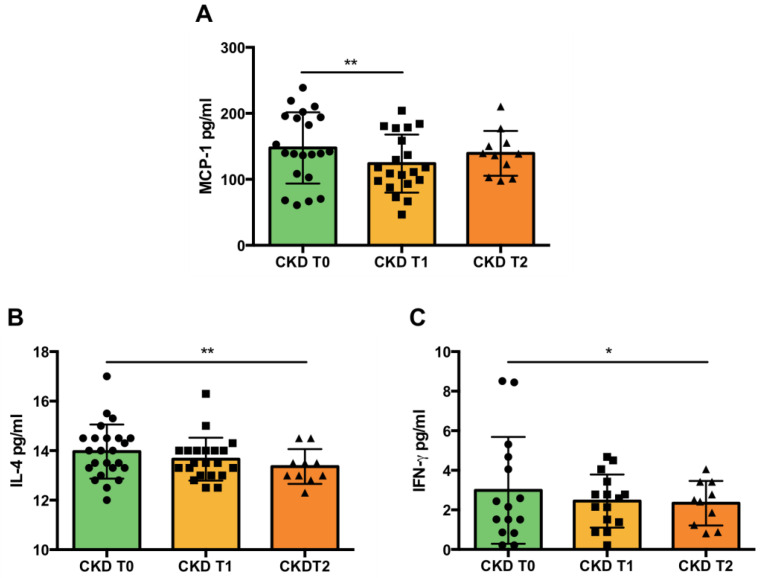
(**A**) Three months curcumin supplementation decreases MCP-1 (CCL-2) plasma levels in CKD patients; ** *p* < 0.01. (**B**,**C**) Six months curcumin supplementation decreases IFN-γ and IL-4 plasma levels in CKD patients; * *p* < 0.05; ** *p* < 0.01.

After three months of curcumin supplementation (T1), there was a significant reduction in the monocyte chemoattractant protein 1 (MCP-1 or CCL-2), as shown in Figure 2A. The curcumin-driven MCP-1 decrease was not confirmed at T2. On the contrary, at T2, a significant decrease in IL-4 and IFN-γ levels was highlighted (Figure 2B,C).

For IL-1β, IL-6, IL-12p70, IL-18, and TNF-α, no statistical differences were found after curcumin supplementation (data not shown).

### 3.4. Lipid Peroxidation

**Figure 3:**
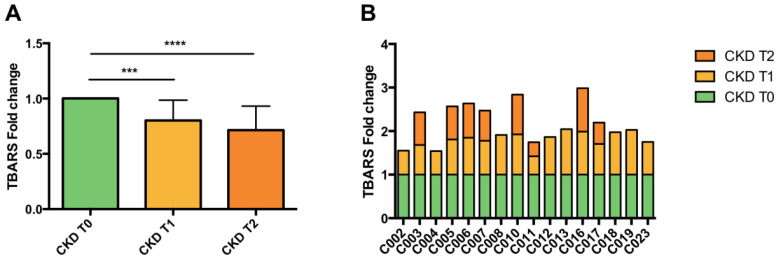
Curcumin supplementation decreases lipid peroxidation in plasma of CKD patients. (**A**) average values of lipid peroxidation (normalized to baseline values, CKD T0) after 3 months (CKD T1) and 6 months (CKD T2) supplementation. (**B**) Examples of intra-individual lipid peroxidation changes after Meriva^®^ supplementation; *** *p* < 0.001; **** *p* < 0.0001.

Raw data showed large inter-individual differences; therefore, the measurements were normalized to baseline values (CKD T0), as presented in Figure 3 A and B. During the first trimester, there was a significant decrease in plasma TBARS levels of CKD patients supplemented with curcumin phytosome (CKD T0 vs. CKD T1; *p* < 0.001), with a mean reduction of 18%. A further decrease in TBARS levels (25%) was observed after six months of supplementation (CKD T0 vs. CKD T2; *p* < 0.0001).

### 3.5. Uremic Toxins

**Figure 4:**
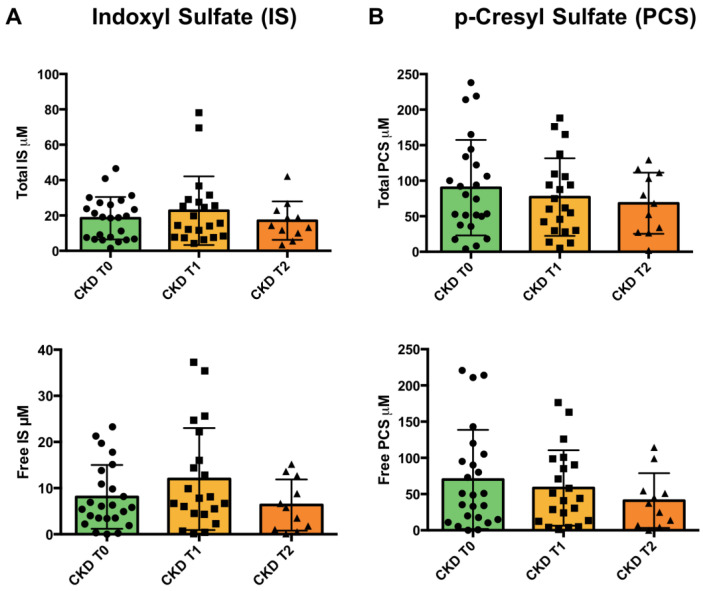
Uremic toxins levels in plasma of CKD patients. (**A**) Total (upper) and free (lower) levels of indoxyl sulfate (IS). (**B**) Total (upper) and free (lower) levels of p-cresyl sulfate (PCS).

The two main uremic toxins known to contribute kidney disease progression are indoxyl sulfate (IS) and p-cresyl sulfate (PCS) [34]. Plasma concentrations of these two molecules were determined at baseline, and after 3 months and 6 months of curcumin supplementation. Although we did not observe a significant decrease in uremic toxins levels at both T1 and T2 (Figure 4A,B), a reduction trend can be noted for total and free PCS levels. 

### 3.6. Gut Microbiota Analysis

After reads alignment and quality filtering, a total of 29,048,198 reads were obtained, with an average of 213,589.691. To avoid biases related to uneven sequencing depth, samples were subsampled to 93,000 reads, according to the amount of sequences in the least abundant sample. 

#### 3.6.1. Bacterial Biodiversity in the Dataset

**Figure 5:**
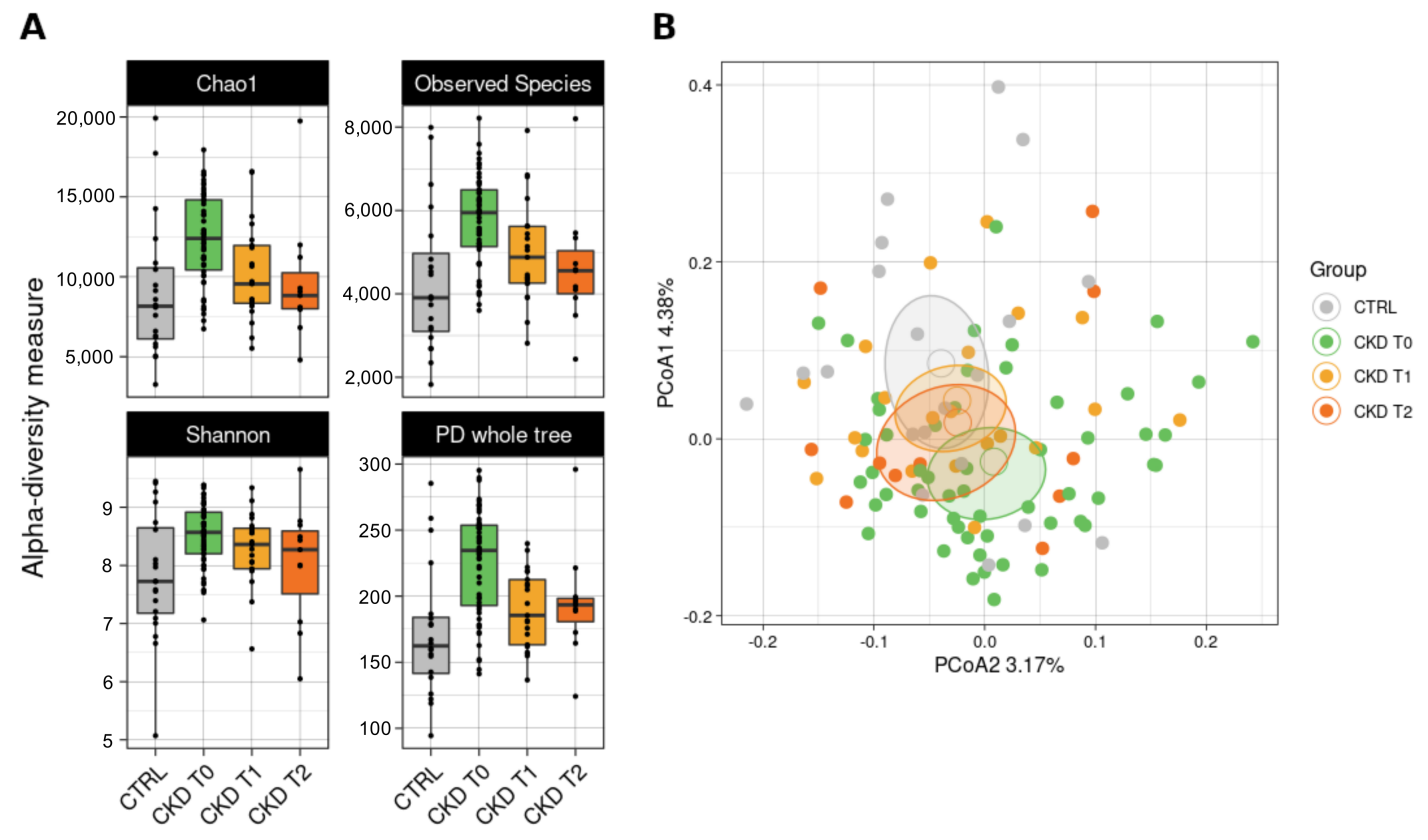
Biodiversity and phylogenetic analysis between cohorts. Bacterial ecological diversity was assessed between CTRL group (grey), CKD group at baseline (green), and CKD groups supplemented with curcumin (CKD T1, yellow; CKD T2, orange). (**A**) Alpha-diversity analysis with Chao1, observed species (OTU count), Shannon index, and Faith’s phylogenetic tree metrics. (**B**) Principal coordinates analysis showing the unweighted Unifrac beta-diversity; the first and second principal coordinates (PCoA1 and PCoA2) are reported.

Initially, we compared the gut microbiota biodiversity of CTRL and CKD groups, before and after curcumin supplementation. CKD patients before curcumin supplementation (T0) showed higher alpha-diversity values compared with CTRL (*p*-values < 0.01, for all metrics). These differences gradually decreased in time after Meriva^®^ intakes (CKD vs. CKD T2, *p* = 0.04 for Chao1 and observed species metrics), leading toward values similar to the CTRL ones (Figure 5A). 

Beta-diversity (Figure 5B) analysis showed a significant divergence between CTRL and CDK at the enrolment (unweighted Unifrac matrix: *p* = 0.023). The Meriva^®^ intake seems to promote a gut microbial shift towards the healthy subject community. 

#### 3.6.2. Taxonomic Composition 

Several taxonomic differences between patients and healthy controls were found at different phylogenetic levels. Taxonomic data at phylum, family, and genus phylogenetic levels are shown in Table 3.

#### 3.6.3. Comparisons with Healthy Subjects

**Figure 6:**
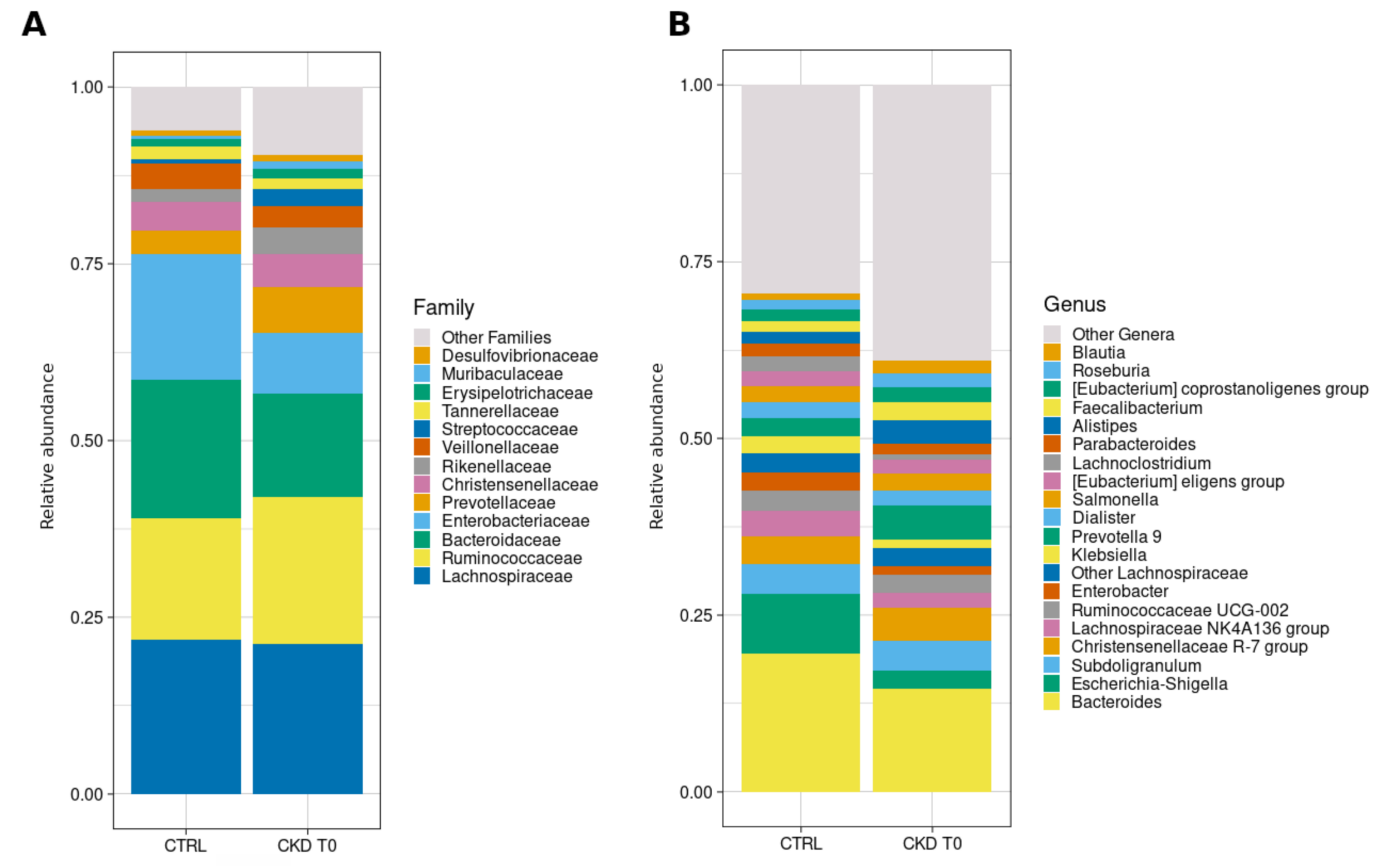
Taxonomic composition of CTRL subjects and CKD patients before curcumin supplementation. Barplots of average microbial relative abundances at family (**A**) and genus (**B**) level for healthy subjects (CTRL) and CKD patients alone (T0).

In order to highlight specific taxa relative abundance variations between healthy subjects and CKD patients, we focused on the patient’s gut composition before Meriva^®^ supplementation (Figure 6). *Bacteroidaceae* family, and *Bacteroides* at genus level, was found significantly lower in CKD patients (19.6% in CTRL, 14.6% in CKD T0, raw *p*-value = 0.037, both at family and at genus levels). Similarly, *Lachnoclostridium* spp. and *Escherichia-Shigella* were depleted in CKD patients (adj *p*-value = 0.018 and *p* = 0.048, respectively). 

**Figure 7:**
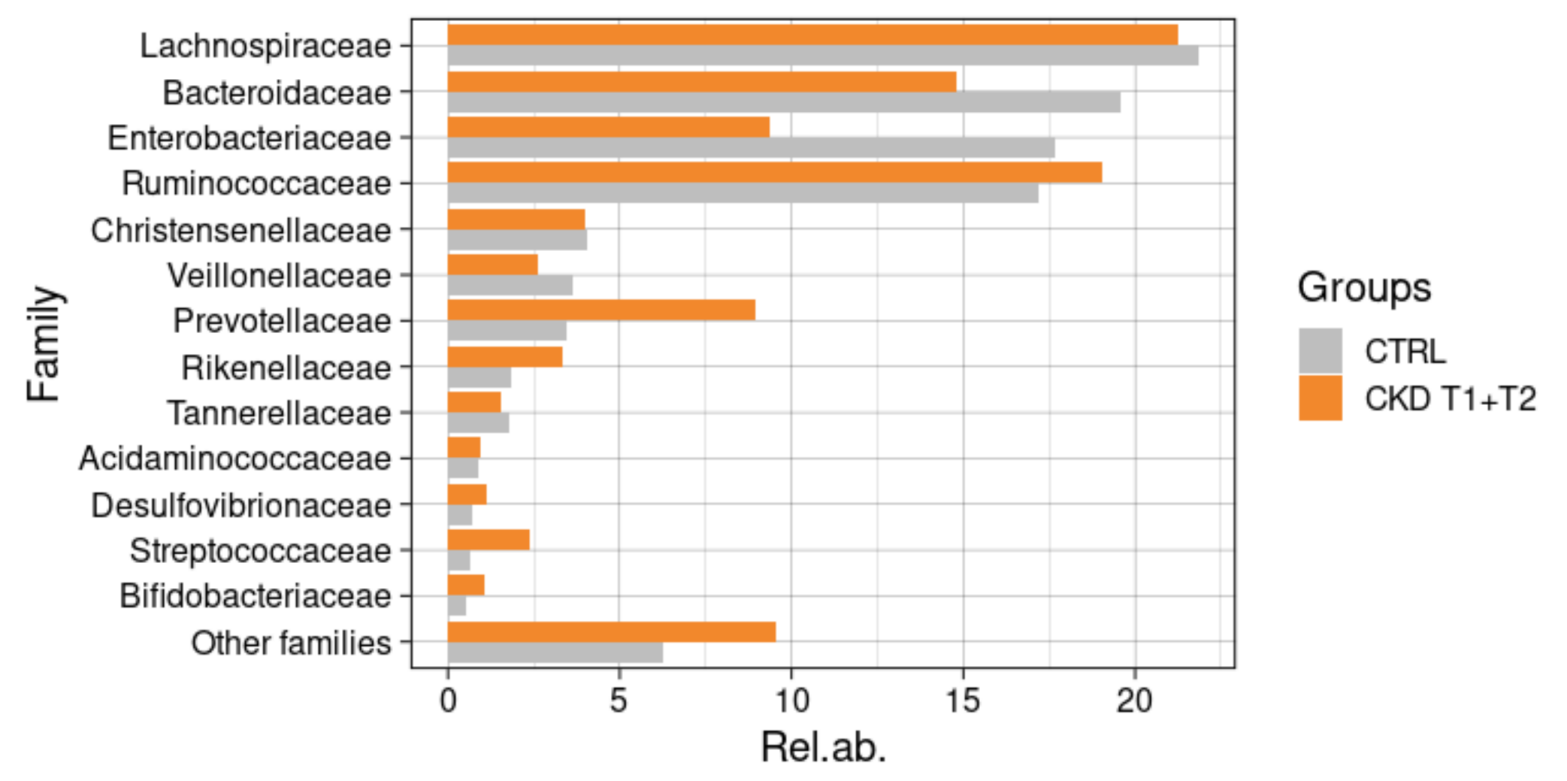
Taxonomic composition of the gut microbiota of healthy subjects and CKD at baseline. Barplots of average microbial relative abundances at family level for the control group (CTRL) and CKD patients after 3 and 6 months of Meriva^®^ supplementation (respectively, CKD T1 and T2) grouped together.

To evaluate Meriva^®^ supplementation effects on CKD gut bacterial community, we compared CTRL subjects and curcumin-treated patients, grouping together CKD T1 and T2 (Figure 7). We observed a decrease in *Verrucomicrobia* (CTRL 0.12% vs. CKD T1 + T2 0.03%; raw *p* = 0.005; adjusted *p* = 0.016) and in *Enterobacteriaceae* relative abundance (*p* = 0.047). Among the *Enterobacteriaceae* family, both *Enterobacter* and *Escherichia-Shigella* genera were found to be depleted compared to healthy controls (adj *p* = 0.033 and *p* = 0.034, respectively). On the other hand, there was a significant increase in the relative abundance of *Lachnoclostridium* (2.04% vs. 0.6%; raw *p* < 0.001) of the *Lachnospiraceae* family (Figure S1).

#### 3.6.4. Taxonomic Changes in CKD Patients during Meriva^®^ Supplementation 

**Figure 8:**
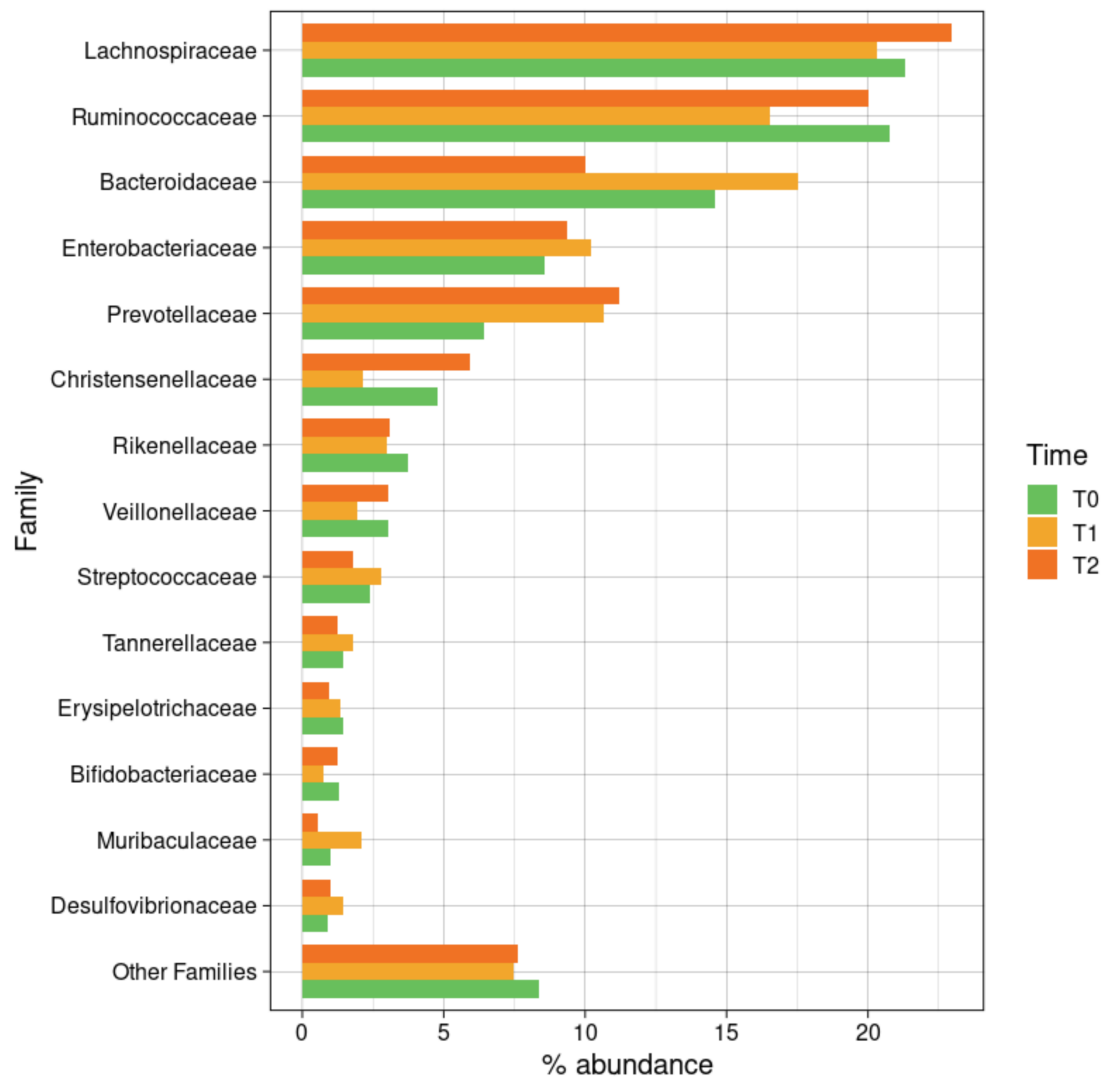
Taxonomic composition of the CKD gut microbiota at family level. Barplots of average microbial relative abundances at family level for CKD patients at baseline (CKD T0) and after 3 and 6 months of Meriva^®^ supplementation (respectively, CKD T1 and T2).

We then focused our attention on curcumin-driven changes in CKD microbiota composition. At phylum level, there was a reduction in the abundance of *Firmicutes* after three months of supplementation compared to untreated CKD (from 47.4% at CKD T0 to 56.7% at CKD T1; *p* = 0.048), as shown in Figure S2. At family level, the relative abundance of *Lactobacillaceae* spp. was found significantly increased at T2 compared to T1 (0.53% vs. 0.15%, raw *p*-value = 0.033) and an interesting increasing trend was observed for *Prevotellaceae*, from baseline to after 6 months of curcumin supplementation (Figure 8). At genus level, *Lachnospira* had a steady increase over time (0.5% at T0, 1.1% at T1, and 2.1% at T2), with a statistically significant difference between baseline and T2 (raw *p*-value = 0.030).

#### 3.6.5. Correlation between Gut Microbiota and Clinical Parameters

**Figure 9:**
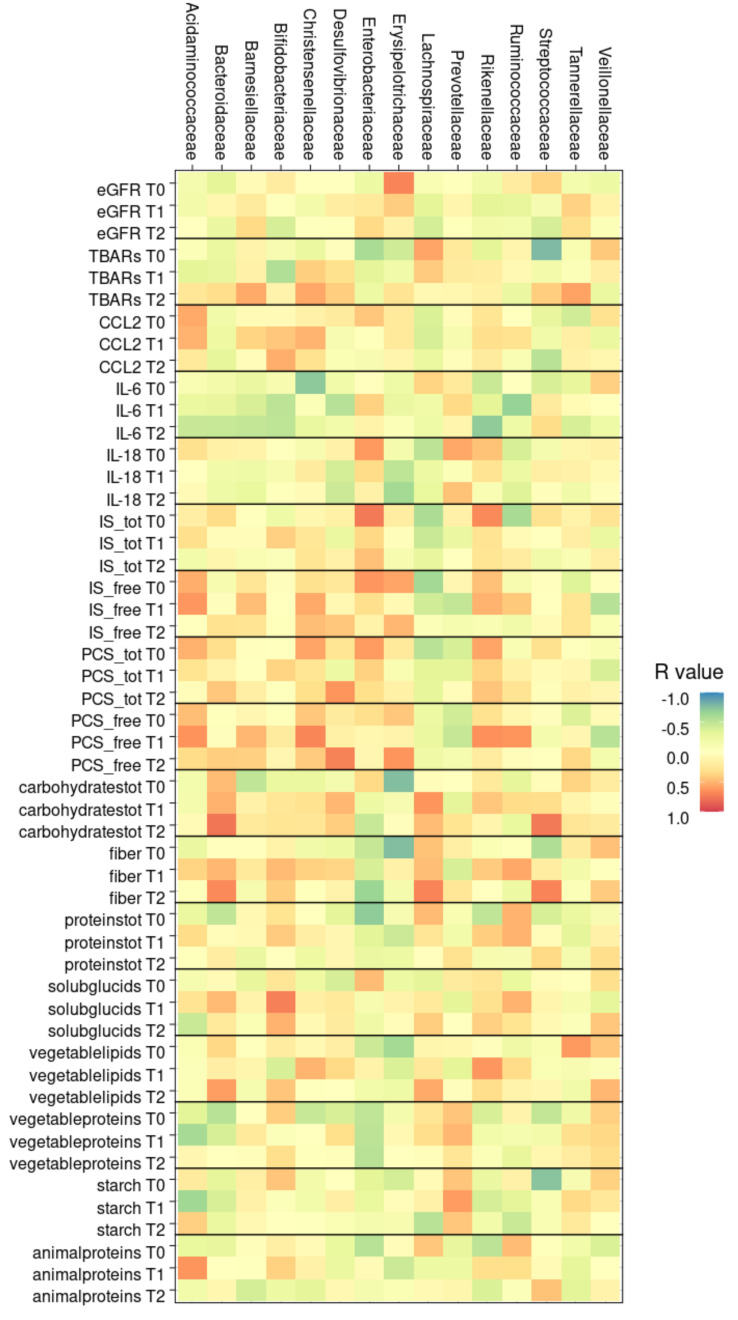
Co-abundant analysis of the main bacterial families and clinical parameters. Co-abundances have been elaborated through Spearman correlation and are shown as R values reporting co-absence (blue) of bacterial and clinical parameter abundances; co-presence of both (red).

A multivariate analysis, to highlight possible influences of gut microbiota changes on clinical parameters and vice versa, was performed by including the most significant and relevant clinical parameters and the most abundant bacterial families (Figure 9) and genera (Figure S3).

Over time, *Enterobacteeriaceae* spp. were found less positively correlated with both total and free indoxyl sulfate (*p*-value = 0.006 for total IS at T0), with PCS, with soluble glucids (*p* = 0.002 at T0), and with eGFR (*p* = 0.021 at T1 and *p* = 0.013 at T2). On the other hand, *Bacteroidaceae* spp. were found statistically related to carbohydrates (*p* = 0.044 at both T1 and T2) and vegetable lipids (*p* = 0.047 at T2), with a higher correlation over the time of curcumin assumption. Notably, although not statistically significant, the *Streptococcaceae* family was found more positively correlated at T2 with carbohydrates and fibers amounts.

## 4. Discussion

In this study, the effects of curcumin phytosome supplementation were investigated in CKD patients through a multifaceted analysis of the nutritional status, dietary intakes, inflammation status, and oxidative stress, together with plasma levels of uremic toxins and gut microbiota composition. 

The investigated clinical parameters showed that, during the six months of supplementation, eGFR did not decline. The majority of CKD patients at baseline were overweight, with an average BMI of 27.19 kg/m^2^. Notably, bioimpedance analysis showed a significant decrease in fat mass and an increasing trend in fat-free mass in the first trimester. This data is in contrast with another study reporting that metabolic alterations caused by CKD usually lead to a decrease in lean mass and consequently to underweight [35]. However, a study conducted in Italy found an average BMI of 27.0 kg/m^2^ in a population of patients with chronic kidney disease, in accordance with our findings [36]. Another study, by Johansen K.L. and colleagues, assessed that 65% of patients with CKD were obese or overweight, with a prevalence of sarcopenia or muscle wasting that ranged from 20% to 44% [37]. 

Nutritional approach is extremely important in CKD because it can limit the progression of renal failure and all associated complications [38]. At the beginning of the study, nutritional counseling was provided to CKD patients. However, the majority of patients were already familiar with the nutritional indications, as they are an integral part of conservative therapy [7]. 

The aim was to recall the importance of lowering protein intake and limiting sodium, phosphorus, and potassium consumption. Results showed a decrease in total daily calories as well as in total carbohydrates and proteins intake. Importantly, a reduction was observed in phosphorus and potassium intake. Patients were more prone to follow our nutritional advice during the first trimester, underlining the importance of monthly surveillance to improve patients’ adherence to the dietetic therapy. 

Curcumin showed an anti-inflammatory effect in lowering plasma cytokines amounts during six months of curcumin supplementation. In particular, a significant decrease of MCP-1 was observed after three months from the beginning of the supplementation. Notably, MCP-1 is overexpressed in patients with CKD and increases inflammation through the recruitment of macrophages involved in atherosclerotic processes [39]. In vitro studies revealed that curcumin can inhibit this cytokine production in human monocytes or alveolar macrophages [40]. Moreover, in vivo studies highlighted the direct effect of curcumin in reducing MCP-1 gene expression in acute small intestinal inflammation or chronic kidney disease, ameliorating intestinal and kidney functions [41,42]. Notably, curcumin supplementation significantly reduced this pro-inflammatory chemokine in humans with metabolic syndrome [43]. 

In our study, curcumin phytosome also induced a reduction in IL-4 and IFN-γ plasma levels after 6 months of supplementation. IL-4 has pleiotropic functions, modulates the immune response by acting on various cell types, such as T lymphocytes, B lymphocytes, macrophages, and endothelial cells [44]. The overproduction is associated with allergic phenomena [45]. Babaei E. and colleagues revealed a significant decrease in IL-4 production in mice with cancer receiving dendrosomal curcumin [46]. The same result was also seen in mice with allergic conjunctivitis after intraperitoneal curcumin injections [47]. A randomized, double-blind, crossover trial investigating serum cytokines levels in obese individuals has shown a decrease in IL-4 amount due to a dietary supplementation with curcuminoids [48], in accordance with our data. 

Regarding IFN-γ, it is implicated in tissue homeostasis, immunomodulation, and inflammation. It is mainly produced by NK (natural killer) cells, which are responsible for the development of kidney damage and promote the onset of CKD [49]. In addition, IFN-γ interferes with the repair process of damaged tubular cells, contributing to the progression of renal disfunction [50]. Moreover, a preclinical study demonstrated the increase of PPARγ and reduction of NFkB, demonstrating the anti-inflammatory effect of curcumin phytosome [21]. To our knowledge, we are reporting for the first time that curcumin supplementation can lower plasma levels of IFN-γ in humans. 

The anti-cytokine effect of curcumin phytosome Meriva® has been demonstrated in different pathological conditions. In particular, curcumin phytosome supplementation induced PPARγ and reduced NFkB in a transgenic mouse model of hepatitis B virus-related hepatocellular carcinoma [21]. PPARγ is a key receptor on cell nucleous that controls the expression of a very large number of genes and plays a fundamental anti-inflammatory role in the immune response through its ability to inhibit the expression of inflammatory cytokines and direct the differentiation of immune cells towards anti-inflammatory phenotypes [21]. Moreover, this curcumin formulation is a promising therapeutic agent in neuroinflammation. A recent study on GFAP-IL6 transgenic mice of chronic neuroinflammation, where IL-6 is overexpressed in the brain, highlighted a positive modulation of neural cells morphology, demonstrating that phytosomal curcumin is able to attenuate the inflammatory pathology [51]. The anti-cytokine effect of curcumin is also currently being studied in Coronavirus disease 2019 (COVID-19). An interesting randomized controlled trial on forty COVID-19 patients supplemented with nano-curcumin showed a significant reduction in IL-1β and IL-6 expression and secretion [52]. The same pro-inflammatory cytokines reduction was demonstrated in patients with osteoarthritis, a pathological condition characterized by chronic inflammation, supplemented with 1 g daily of phytosomal curcumin for 8 months [53].

Lipid peroxidation analysis showed an average decrease of 18% after three months from the start of the study and an average decrease of 25% at the end of six months of supplementation. Numerous in vitro studies already explained how curcumin acts with antioxidant activity by limiting oxidative processes, reducing circulating ROS and lipid peroxidation phenomena. Mohamedain M. Mahfouz and colleagues showed that the presence of curcumin decreases conjugated diene and lipid peroxides in oxidized LDL isolated from human plasma [54]. Another study performed by the University of Amsterdam observed that curcumin suppresses the MAP-kinase pathway in human fetal astrocytes and reduces reactive oxygen species in neuronal cells [55]. Sadeghi A. and colleagues described the role of curcumin in ameliorating the inflammatory responses stimulated by palmitate in muscular cells: curcumin repressed the phosphorylation of IKKα, IKKβ, and JNK and decreased ROS levels [56]. Other preclinical studies showed an increased amount of antioxidant enzymes after curcumin supplementation [57,58,59]. However, the antioxidant effects of curcumin have not been extensively documented on human individuals. According to our results on lipid peroxidation, a reduction in TBARS levels was observed in patients with diabetes mellitus after curcumin supplementation in a randomized, parallel-group, placebo-controlled study [60]. The antioxidant effect of curcumin was seen also in a single-blind, randomized study where twenty patients with tropical pancreatitis showed a significant reduction in the erythrocyte MDA levels after curcumin therapy [61].

Then, we evaluated the two main uremic toxins known to contribute to kidney disease progression: indoxyl sulfate (IS) and p-cresyl sulfate (PCS) [34]. IS and PCS are produced by human colon microorganisms from dietary amino acids, are directly responsible for the progression of renal damage, and increase cardiovascular risks [62]. Plasma concentrations of these two molecules did not significantly change after 3 or 6 months of curcumin supplementation. Noteworthy, plasma concentrations of IS and PCS did not increase in the supplemented group, supporting the stability observed in the clinical parameters of the enrolled CKD population. Usually, there is an increment in uremic toxins levels as the renal damages get worse, but this was not observed after supplementation. Interestingly, a reduction trend can be noted for total and free PCS levels, after both 3 and 6 months of curcumin phytosome supplementation. 

In a recent study by Salarolli et al., the oral supplementation of curcumin (2.5 g of turmeric for three months) seemed to reduce PCS plasma levels in hemodialysis patients [63]. 

The presence of dysbiosis in CKD patients leads to an increased growth of microorganisms with proteolytic enzymes involved in the metabolism of uremic toxins [64]. 

Surprisingly, the present study found higher alpha-diversity values in CKD groups compared with CTRL subjects, age- and sex-matched. Noteworthy, CKD patients followed specific nutritional advice, whereas the CTRL group was on a free diet. A recent systematic review investigated the alteration of gut microbiota in CKD compared to healthy subjects [65]: only 33.3% of the studies showed higher richness in CKD subjects, among which we found an Italian study that reported our same result [66]. 

In particular, in our study, *Bacteroidaceae* family was found significantly lower in CKD patients, and *Bacteroides* followed the same abundances and statistics at genus level. *Lachnoclostridium* spp. were found deeply reduced in CKD patients, as well as *Escherichia-Shigella*, which was found present at 8.4% of relative abundance in healthy subjects and at 2.5% in CKD patients. To better understand the effects of Meriva^®^ supplementation on the CKD gut microbiota, we performed a comparison of CTRL subjects and patients after the months of curcumin intake, grouping together CKD T1 and T2. At phylum level, in Meriva^®^ supplementation there was a significantly lower presence of *Verrucomicrobia,* whereas, at family level, *Enterobacteriaceae* were found significantly lower in CKD patients supplemented with curcumin, reflected in an analogous lower abundance of the *Enterobacter* genus in the same cohort. Of the same family, at genus level, *Escherichia-Shigella* was found reduced as well, while there was a significant higher presence of *Lachnoclostridium* of the *Lachnospiraceae* family. Notably, at family level, *Lactobacillaceae* spp. were found significantly higher at T2 compared with T1 and an interesting increasing trend was observed in the *Prevotellaceae* group, from baseline to after 6 months of curcumin supplementation. 

Saccharolytic and butyrate-producing bacteria (*Prevotella, F. prausnitzii, Roseburia*) seem to play an important role in the maintenance of gut barrier function. In fact, butyrate promotes colon motility, reduces inflammation, increases visceral vascularization, inhibits tumor cell progression, and induces differentiation of T-regulatory cells [67].

Some studies described how curcumin helps to maintain the integrity of the intestinal barrier and its permeability, promoting a better composition of the intestinal microbiota. This activity was studied mainly in mice. It was observed that nanoparticle curcumin improves gut microbiota structure and mucosal permeability in mice with colitis [68]. A study by Feng W. et al. showed that mice fed a high-fat-diet and supplemented with curcumin decreased hepatic fat deposition and ameliorated gut microbiota composition [69]. 

Our study has a design limitation regarding the small sample size and the high drop-out rates, not related to curcumin supplementation. However, we believe that CKD groups were in-depth characterized, with promising results that should be confirmed in a placebo-controlled trial.

## 5. Conclusions

Our pilot study demonstrated the promising benefits of curcumin supplementation in chronic kidney disease. In particular, curcumin phytosome significantly reduced plasma pro-inflammatory mediators (MCP-1, IFN-γ, and IL-4) and lipid peroxidation. It is noteworthy that plasma concentrations of uremic toxins did not increase in the supplemented group, supporting the stability observed on the clinical parameters of the enrolled CKD population. Regarding gut microbiota analysis, after 6 months of curcumin supplementation, CKD’s alpha-diversity showed a significant trend toward values similar to the healthy ones. At phylum level, there was a significantly lower presence of *Escherichia-Shigella*, while there was a significant higher presence of *Lachnoclostridium*. Notably, at family level, *Lactobacillaceae* spp. were found significantly higher in the last 3 months of supplementation.

Regarding tolerability, no adverse events were observed in the supplemented group, confirming the good safety profile of curcumin phytosome after long-term administration. 

## Figures and Tables

**Table 1:** Cohort characteristics. Data are reported as median (IQR).

	CTRL (*n* = 20)	CKD T0 (*n* = 24)	CKD T1 (*n* = 21)	CKD T2 (*n* = 11)	*p*-Value
Gender	M: 60% (*n* = 12) F: 40% (*n* = 8)	M: 58% (*n* = 14) F: 42% (*n* = 10)	M: 57% (*n* = 12) F: 43% (*n* = 9)	M: 45.5% (*n* = 5) F: 54.5% (*n* = 6)	n.s.
Age (Y)	74 (68.5–78.7)	72 (67.5–78.8)	72 (66–77)	72 (67–81)	n.s.
Height (m)	1.68 (1.58–1.75)	1.65 (1.62–1.71)	1.65 (1.62–1.70)	1.64 (1.60–1.71)	n.s.
Weight (kg)	75 (66–80)	77.5 (63.3–83.0)	79.0 (64.7–81.0)	80.0 (64.0–85.0)	n.s.
BMI (kg/m^2^)	25.20 (22.50–30.70)	27.40 (22.62–31.50)	27.53 (23.42–30.35)	28.04 (22.68–33.06)	n.s.
Waist circumference (cm)	99.0 (90.5–111.0)	102.0 (91.0–110.5)	103.0 (97.0–108.0)	99.5 (93.0–110.0)	n.s.
Bioimpedance					
TBW (l)	37.7 (29.9–44.1)	36.8 (33.5–42.2)	38.1 (34.8–40.6)	37.2 (35.3–45.0)	*** CKD T0 vs. CKD T1
ECW (I)	17.7 (14.2–19.9)	17.7 (15.5–19.5)	18.0 (16.1–20.0)	17.0 (16.1–19.2)	n.s.
ICW (I)	19.8 (15.8–23.0)	19.5 (17.5–21.6)	20.0 (18.0–22.1)	20.0 (19.1–22.9)	n.s.
FFM (%)	54.8 (46.0–60.3)	52.7 (46.6–59.5)	54.6 (49.2–66.2)	53.6 (46.2–67.9)	n.s.
FM (%)	31.4 (25.1–38.4)	31.8 (28.2–37.3)	30.9 (24.5–34.4)	33.0 (21.7–37.6)	*** CKD T0 vs. CKD T1
Clinical parameters					
Creatininemia (mg/dL)	0.80 (0.70–1.00)	1.96 (1.46–2.73)	1.90 (1.57–2.80)	1.45 (1.48–2.50)	*** CTRL vs. CKD T0 *** CTRL vs. CKD T1 *** CTRL vs. CKD T2
GFR (ml/min/1.73 m^2^)	83.0 (70.0–91.0)	33.5 (22.1–39.8)	27.2 (21.0–45.5)	35.7 (24.0–45.0)	*** CTRL vs. CKD T0 *** CTRL vs. CKD T1 *** CTRL vs. CKD T2
Azotemia (mg/dL)	n.d.	60.0 (30.0–90.0)	46.5 (32.5–63.5)	35.0 (30.0–40.0)	n.s.
Sodium (mEq/L)	140.0 (138.5–141.5)	142.3 (139.3–143.8)	141.9 (140.0–143.5)	143.0 (140.7–143.8)	n.s.
Potassium (mEq/L)	4.40 (4.25–5.10)	4.60 (4.46–5.08)	4.60 (4.25–4.80)	4.55 (4.37–4.77)	n.s.
Phosphorus (mg/dL)	n.d.	3.65 (3.20–4.00)	3.50 (3.00–4.00)	3.30 (3.10–4.10)	n.s.
Calcium (mg/dL)	n.d.	9.60 (9.31–9.99)	9.60 (9.45–9.99)	9.48 (9.26–9.59)	n.s.

CTRL, control group; CKD T0, chronic kidney disease group at baseline; CKD T1, chronic kidney disease group after 3 months of supplementation; CKD T2, chronic kidney disease group after 6 months of supplementation; IQR, interquartile range; Y, years; BMI, body mass index; TBW, total body water; ECW, extracellular water; ICW, intracellular water; FFM, fat-free mass; FM, fat mass; GFR, glomerular filtration rate; n.d., not determined; n.s., not significant; *** *p* < 0.0001.

**Table 2:** Analysis of the differences in food consumption between the CTRL and the CKD groups and effect of the nutritional counseling on the food consumption of CKD patients. Data are reported as median (IQR).

	CTRL (*n* = 20)	CKD T0 (*n* = 24)	CKD T1 (*n* = 21)	CKD T2 (*n* = 11)	*p*-Value
Caloric intake (kcal/day)	1788 (1438–2226)	1839 (1399–2520)	1540 (1195–1871)	1270 (1131–2612)	* CKD T0 vs. CKD T1
Total carbohydrates (g/day)	154.4 (105.8–230.2)	215.9 (169.4–267.7)	160.6 (136.7–235.6)	128.0 (109.9–200.1)	* CTRL vs. CKD T0 ** CKD T0 vs. CKD T1 *** CKD T0 vs. CKD T2
Starch (g/day)	93.34 (62.51–156.00)	118.80 (67.72–163.20)	89.56 (58.20–120.0)	64.77 (27.36–85.33)	* CKD T0 vs. CKD T1 ** CKD T0 vs. CKD T2
Soluble glucids (g/day)	52.06 (36.70–72.27)	64.62 (46.90–92.12)	56.32 (36.50–79.68)	50.47 (36.03–60.06)	* CKD T0 vs. CKD T2
Fibers (g/day)	12.85 (9.80–18.58)	16.97 (14.49–20.35)	13.95 (11.41–16.07)	13.91 (10.89–19.64)	* CKD T0 vs. CKD T1
Total lipids (g/day)	52.72 (43.05–64.90)	57.15 (41.01–76.42)	49.29 (42.35–69.62)	49.82 (36.49–71.93)	n.s.
Animal lipids (g/day)	23.33 (16.59–33.67)	28.95 (18.07–38.84)	28.84 (19.47–35.98)	21.57 (14.24–30.28)	n.s.
Plant lipids (g/day)	27.58 (24.24–38.82)	28.49 (20.14–33.55)	22.04 (17.95–26.55)	21.73 (15.73–32.62)	n.s.
Total proteins (g/day)	59.18 (43.96–75.47)	60.08 (49.39–70.34)	49.17 (42.92–59.74)	40.66 (34.31–73.92)	* CKD T0 vs. CKD T1
Animal proteins (g/day)	38.79 (31.37–50.88)	35.60 (24.17–47.05)	33.21 (24.43–40.70)	31.60 (22.14–40.58)	n.s.
Plant proteins (g/day)	19.01 (12.54–27.40)	23.16 (14.80–28.23)	16.37 (13.28–21.52)	13.67 (10.09–17.09)	*** CKD T0 vs. CKD T1 *** CKD T0 vs. CKD T2
Phosphorus (mg/day)	997.4 (718.1–1216.0)	862.4 (756.2–1047.0)	813.8 (665.2–894.3)	684.2 (609.5–874.2)	* CTRL vs. CKD T1 ** CKD T0 vs. CKD T1
Potassium (g/day)	2.38 (1.71–3.66)	2.27 (1.78–2.75)	1.89 (1.60–2.39)	1.98 (1.58–2.28)	* CTRL vs. CKD T1 ** CKD T0 vs. CKD T1

CTRL, control group; CKD T0, chronic kidney disease group at baseline; CKD T1, chronic kidney disease group after 3 months of supplementation; CKD T2, chronic kidney disease group after 6 months of supplementation; IQR, interquartile range; * *p* < 0.05; ** *p* < 0.01; *** *p* < 0.001.

**Table 3:** Taxonomic abundances and significances. Average relative abundance and standard deviation at phylum, family, and genus phylogenetic levels.

Phylum	Family	Genus	CTRL	CKD T0	CKD T1	CKD T2	*p*-Value
*Firmicutes*			50.43 (20.54)	56.67 (15.24)	47.46 (12.01)	57.46 (18.61)	0.0428 (T0-T1)
	*Lachnospiraceae*		21.84 (10.43)	21.30 (10.00)	20.32 (8.38)	22.96 (15.40)	0.014 (CTRL-T2); 0.033 (T1-T2)
		*Other Lachnospiraceae*	2.62 (2.54)	2.51 (2.82)	3.24 (2.26)	1.42 (1.40)	
		*Lachnospiraceae NK4A136 group*	3.65 (4.96)	2.19 (2.64)	3.46 (3.65)	2.75 (2.35)	
		*Roseburia*	1.43 (1.55)	2.04 (2.90)	1.57 (2.43)	4.41 (3.03)	
		*[Eubacterium] eligens group*	2.13 (2.31)	2.00 (1.40)	1.41 (1.67)	1.72 (2.34)	
		*Blautia*	0.93 (0.80)	1.83 (0.57)	1.11 (0.82)	0.35 (3.71)	
	*Ruminococcaceae*		17.20 (12.30)	20.75 (10.80)	16.53 (8.06)	20.00 (10.11)	
		*Subdoligranulum*	4.14 (5.40)	4.23 (2.45)	5.38 (7.41)	3.71 (7.10)	
		*Ruminococcaceae UCG-002*	2.84 (5.02)	2.48 (3.30)	2.76 (2.65)	3.12 (3.13)	
		*Faecalibacterium*	1.58 (2.26)	2.46 (2.12)	2.63 (2.87)	2.01 (4.77)	
		*Ruminococcus 2*	0.48 (0.57)	2.39 (1.66)	2.26 (3.35)	2.33 (4.10)	
		*[Eubacterium] coprostanoligenes group*	1.57 (1.92)	2.07 (1.12)	1.19 (0.76)	1.04 (2.08)	0.0453 (T0-T1)
	*Christensenellaceae*		4.03 (5.35)	4.77 (6.02)	2.14 (2.24)	5.95 (12.73)	
		*Christensenellaceae R-7 group*	3.93 (5.26)	4.66 (2.04)	5.66 (12.08)	2.20 (5.97)	
	*Veillonellaceae*		3.61 (7.28)	3.02 (4.39)	1.93 (2.66)	3.02 (2.59)	
		*Dialister*	2.31 (6.57)	2.10 (1.15)	2.24 (2.64)	2.35 (4.23)	
	*Streptococcaceae*		0.65 (0.85)	2.37 (3.85)	2.77 (4.97)	1.78 (2.64)	
		*Streptococcus*	0.64 (0.84)	2.36 (2.76)	1.76 (2.64)	4.97 (3.84)	
	*Erysipelotrichaceae*		1.00 (1.83)	1.44 (1.41)	1.36 (1.55)	0.97 (0.84)	
	*Acidaminococcaceae*		0.88 (1.44)	1.11 (1.69)	1.02 (1.46)	0.48 (0.71)	
*Bacteroidetes*			28.01 (15.40)	28.90 (14.45)	36.80 (12.08)	27.68 (16.73)	
	*Muribaculaceae*		0.47 (1.06)	0.97 (2.55)	2.09 (6.01)	0.57 (1.64)	
	*Barnesiellaceae*		0.62 (0.80)	0.93 (1.28)	1.03 (0.95)	1.07 (1.65)	
	*Bacteroidaceae*		19.58 (15.17)	14.59 (11.04)	17.55 (11.52)	10.01 (7.27)	0.037 (CTRL-T2)
		*Bacteroides*	19.57 (15.17)	14.586 (17.55)	10.01 (7.27)	11.52 (11.04)	
	*Prevotellaceae*		3.44 (7.46)	6.42 (10.04)	10.68 (13.90)	11.19 (18.77)	
		*Prevotella 9*	2.48 (7.54)	4.76 (7.20)	9.32 (17.86)	12.22 (9.25)	
	*Rikenellaceae*		1.83 (1.40)	3.75 (3.92)	2.97 (3.58)	3.09 (4.04)	
		*Alistipes*	1.67 (1.34)	3.44 (2.24)	2.07 (1.99)	1.69 (3.85)	
	*Tannerellaceae*		1.79 (1.83)	1.46 (0.93)	1.78 (1.08)	1.25 (1.11)	
		*Parabacteroides*	1.79 (1.83)	1.46 (1.78)	1.25 (1.10)	1.081 (0.93)	
*Proteobacteria*			19.56 (23.55)	10.48 (11.26)	13.27 (14.16)	11.66 (18.12)	
	*Enterobacteriaceae*		17.68 (24.35)	8.58 (11.54)	10.21 (14.29)	9.34 (18.94)	
		*Escherichia-Shigella*	8.44 (15.15)	2.54 (3.41)	5.20 (10.86)	5.64 (5.12)	
		*Salmonella*	2.25 (3.62)	2.44 (2.85)	2.09 (4.28)	3.88 (2.83)	
		*Klebsiella*	2.52 (5.55)	1.32 (1.70)	0.53 (1.30)	3.88 (2.17)	
	*Desulfovibrionaceae*		0.72 (0.93)	0.89 (0.66)	1.45 (1.24)	0.99 (1.10)	0.020 (CTRL-T1)
*Actinobacteria*			1.00 (1.02)	2.36 (2.90)	1.35 (1.24)	1.95 (2.85)	0.0361 (CTRL-T0)
	*Bifidobacteriaceae*		0.51 (0.67)	1.30 (1.88)	0.75 (1.08)	1.27 (2.73)	
	*Coriobacteriaceae*		0.15 (0.25)	0.52 (1.11)	0.27 (0.31)	0.25 (0.42)	
*Verrucomicrobia*			0.12 (0.12)	0.03 (0.09)	0.09 (0.04)	0.11 (0.01)	

CTRL, healthy subjects; CKD T0, CKD patients at baseline; CKD T1 and CKD T2, CKD patients after 3 and 6 months of Meriva^®^ supplementation, respectively. Only adjusted *p*-values (Bonferroni correction) are reported; *p*-values < 0.05 were considered significant.

## Data Availability

The data presented in this study are available on request from the corresponding author.

## Supplementary Materials

The following are available online at https://www.mdpi.com/article/10.3390/nu14010231/s1, Figure S1: Taxonomic composition of the gut microbiota of healthy subjects and CKD at baseline; Figure S2: Taxonomic composition of the CKD gut microbiota at family level; Figure S3: Co-abundant analysis of the main bacterial families and clinical parameters.
