# Supplementary material for: Curcumin Supplementation (Meriva®) Modulates Inflammation, Lipid Peroxidation and Gut Microbiota Composition in Chronic Kidney Disease
Source: Nutrients. 2022 Jan 5;14(1):231. doi: 10.3390/nu14010231 (PMC8747135; doi:10.3390/nu14010231)
Supplement: Supplementary file 1 [file nutrients-14-00231-s001.zip › nutrients-1521804-supplementary-done.pdf]

nutrients

Curcumin Supplementation (Meriva®) Modulates Inflammation, Lipid Peroxidation and Gut Microbiota Composition in Chronic Kidney Disease

Francesca Pivari [1,*,†], Alessandra Mingione [1,†], Giada Piazzini [1], Camilla Ceccarani [2], Emerenziana Ottaviano [3], Caterina Brasacchio [4], Michele Dei Cas [5], Margherita Vischi [6], Mario Gennaro Cozzolino [4,6], Paolo Fogagnolo [4,7], Antonella Riva [8], Giovanna Petrangolini [8], Luigi Barrea [9], Laura Di Renzo [10], Elisa Borghi [3], Paola Signorelli [1], Rita Paroni [5] and Laura Soldati [4,*]

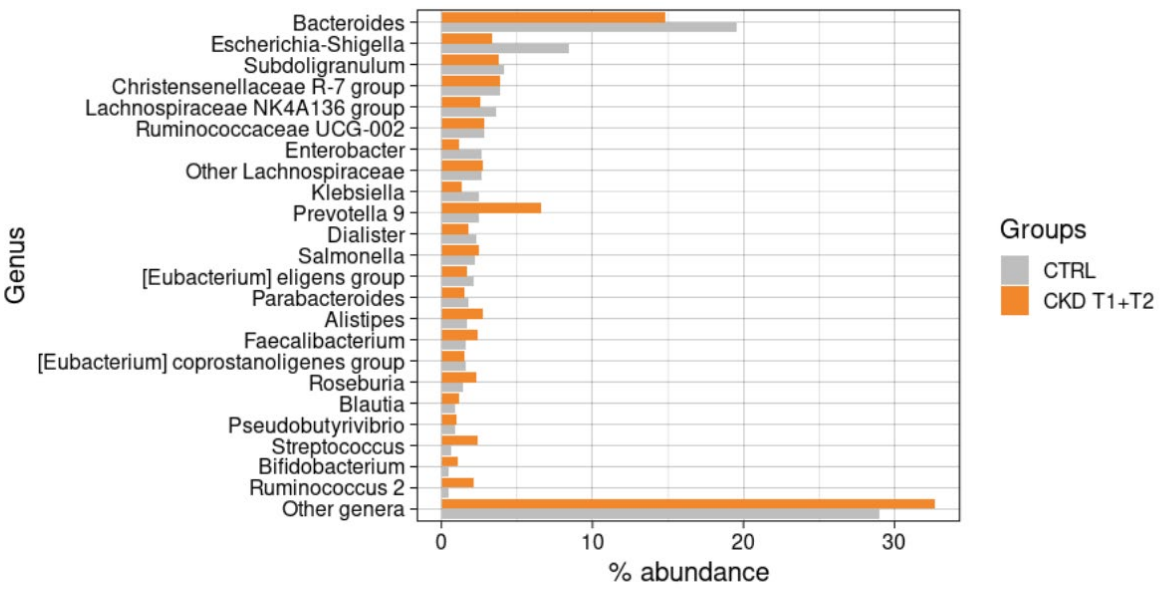

Figure S1. Taxonomic composition of the gut microbiota of healthy subjects and CKD at baseline.

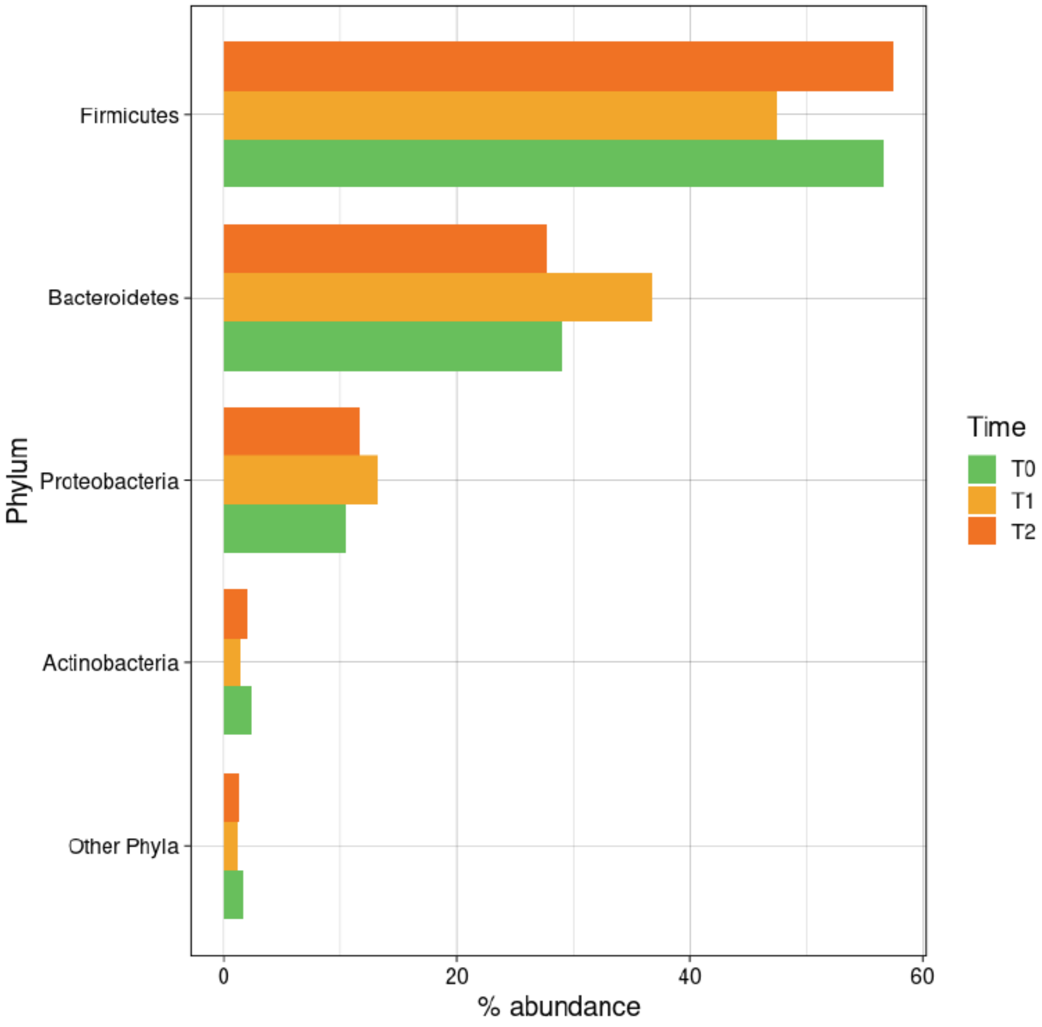

Figure S2. Taxonomic composition of the CKD gut microbiota at family level.

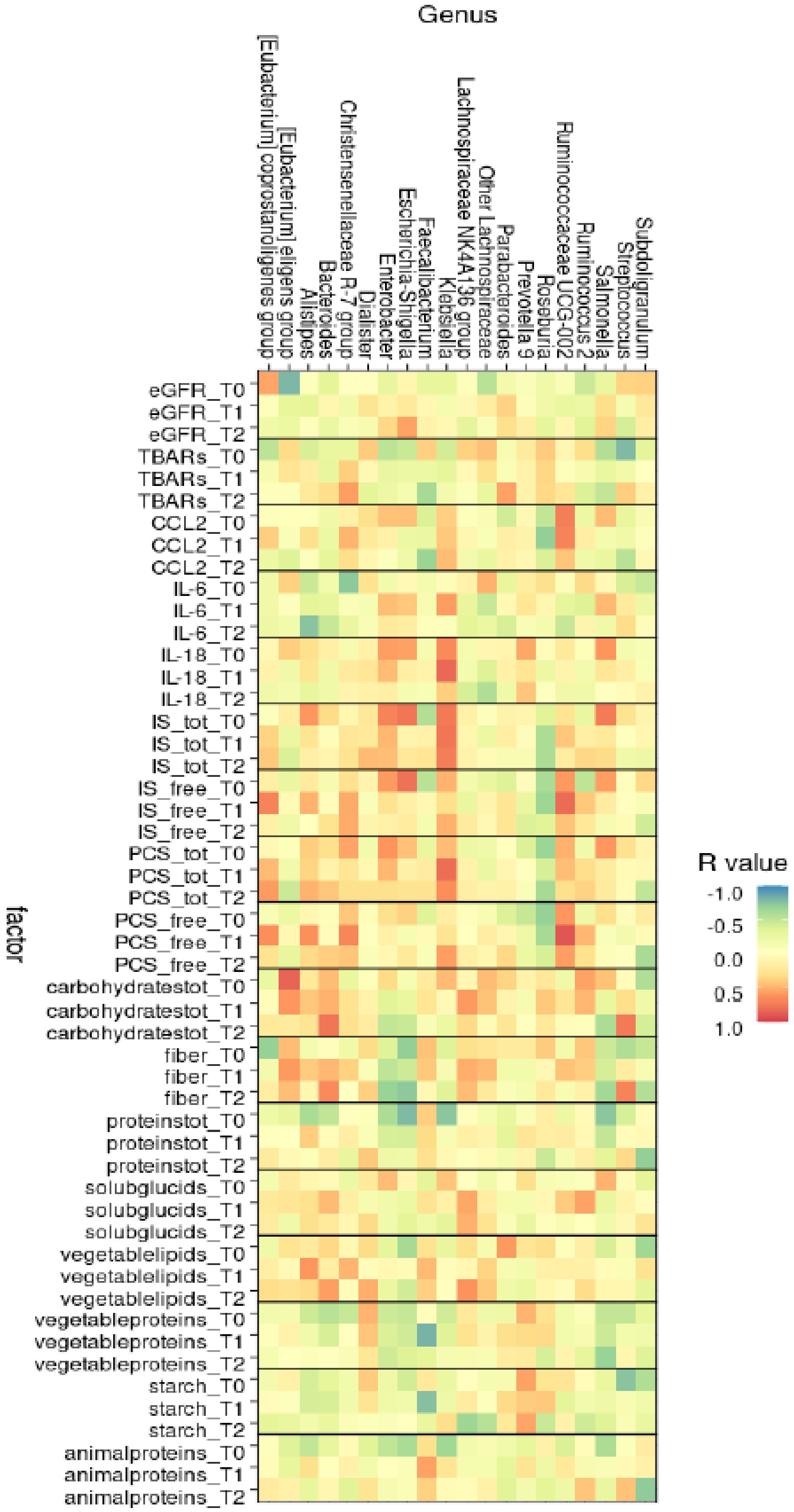

Figure S3. Co-abundant analysis of the main bacterial families and clinical parameters.
